# *Ureaplasma urealyticum* Causes Hyperammonemia in an Experimental Immunocompromised Murine Model

**DOI:** 10.1371/journal.pone.0161214

**Published:** 2016-08-18

**Authors:** Xiaohui Wang, Melissa J. Karau, Kerryl E. Greenwood-Quaintance, Darci R. Block, Jayawant N. Mandrekar, Scott A. Cunningham, Robin Patel

**Affiliations:** 1 Division of Clinical Microbiology, Department of Laboratory Medicine and Pathology, Mayo Clinic, Rochester, Minnesota, United States of America; 2 Division of Clinical Core Laboratory, Department of Laboratory Medicine and Pathology, Mayo Clinic, Rochester, Minnesota, United States of America; 3 Department of Health Sciences Research, Mayo Clinic, Rochester, Minnesota, United States of America; 4 Division of Infectious Diseases, Department of Medicine, Mayo Clinic, Rochester, Minnesota, United States of America; US Geological Survey, UNITED STATES

## Abstract

Hyperammonemia syndrome is an often fatal complication of lung transplantation which has been recently associated with *Ureaplasma* infection. It has not been definitely established that *Ureaplasma* species can cause hyperammonemia. We established a novel immunocompromised murine model of *Ureaplasma urealyticum* infection and used it to confirm that *U*. *urealyticum* can cause hyperammonemia. Male C3H mice were pharmacologically immunosuppressed with mycophenolate mofetil, tacrolimus and oral prednisone for seven days, and then challenged intratracheally (IT) and/or intraperitoneally (IP) with 10^7^ CFU *U*. *urealyticum* over six days, while continuing immunosuppression. Spent *U*. *urealyticum-*free U9 broth was used as a negative control, with uninfected immunocompetent mice, uninfected immunosuppressed mice, and infected immunocompetent mice serving as additional controls. Plasma ammonia concentrations were compared using Wilcoxon ranks sum tests. Plasma ammonia concentrations of immunosuppressed mice challenged IT/IP with spent U9 broth (n = 14) (range 155–330 μmol/L) were similar to those of normal mice (n = 5), uninfected immunosuppressed mice (n = 5), and *U*. *urealyticum* IT/IP challenged immunocompetent mice (n = 5) [range 99–340 μmol/L, p = 0.60]. However, immunosuppressed mice challenged with *U*. *urealyticum* IT/IP (n = 20) or IP (n = 15) had higher plasma ammonia concentrations (range 225–945 μmol/L and 276–687 μmol/L, respectively) than those challenged IT/IP with spent U9 broth (p<0.001). *U*. *urealyticum* administered IT/IP or IP causes hyperammonemia in mice pharmacologically immunosuppressed with a regimen similar to that administered to lung transplant recipients.

## Introduction

Hyperammonemia syndrome (HS) is a rare and potentially fatal complication of lung transplantation [1–8]. Case series show that this syndrome may occur in as many as 4.1% of lung transplant recipients, with 67% of affected patients dying within 30 days of lung transplantation [3]. Until recently, the etiology of HS was unclear, with potential etiologies suggested in individual cases to include hepatic glutamine synthetase deficiency [7], and disseminated *Mycoplasma hominis* infection [6]. Recently, using PCR and specialized culture, we found evidence of *Ureaplasma* infection in lung transplant recipients with HS [8]. However, causality had not yet been established. Herein, we established a new experimental pharmacologically immunosuppressed murine model of *U*. *urealyticum* infection and used it to show that *U*. *urealyticum* can cause hyperammonemia. The immunosuppression regimen studied was designed to mimic that administered to lung transplant recipients.

## Methods

### Ethics Statement

This study was carried out in accordance with the recommendations in the Guide for the Care and Use of Laboratory Animals of the National Institutes of Health, and was approved by Mayo Clinic Institutional Animal Care and Use Committee (protocol number: A8115). Mayo Clinic is AAALAC accredited (000717), registered with the USDA (41-R-0006), and has an Assurance with OLAW (A3291-01). Mice were housed in a biosafety level 2, specific-pathogen-free, AAALAC-accredited facility, where sentinel mice were tested quarterly for murine pathogens; tested mice were negative for murine pathogens throughout the course of this study. Mice had unrestricted access to irradiated rodent food (LabDiet formula 5053) and water. The housing room was environmentally controlled (temperature 68–74°F, relative humidity 30–70%, 12:12-hour light:dark cycle). All efforts were made to minimize suffering. Mice were monitored twice daily, and anesthetized mice were monitored until awake. Animals were monitored for decreased activity, decreased body temperature, hunched stature, distress, and inability to eat and drink; if these findings were severe, animals were euthanized.

### Microorganism

*U*. *urealyticum* ATCC 27618 (American Type Culture Collection, Manassas, VA) was used to challenge the mice. For inoculum preparation, *U*. *urealyticum* was cultivated in U9 broth (Hardy Diagnostics, Santa Maria, CA) at 37°C in air until a color change was observed (~7 hours). The culture broth was centrifuged at 4,000 rpm for 30 minutes (to concentrate *U*. *urealyticum*). Cells were resuspended in spent U9 broth to prepare final concentrations of 2x10^8^ CFU/mL. Fresh inoculum was immediately administered. For morphological identification and quantitation, *U*. *urealyticum* was plated onto A8 agar (Hardy Diagnostics) and incubated anaerobically at 37°C for five days. Colonies were enumerated by 100X microscopy.

### Immunosuppressive Agents

Mice were pharmacologically immunosuppressed using intraperitoneal (IP) mycophenolate mofetil (90 mg/kg) (Cellcept Intravenous, Roche Laboratories, Inc., Nutley, NJ), IP tacrolimus (1.2 mg/kg) (Prograf, Astellas Pharma US, Inc., Northbrook, IL), and oral prednisone (6 mg/kg) (Prednisone Intensol, Roxane Laboratories, Inc., Columbus, OH) administered daily for seven days prior to challenge with *U*. *urealyticum* and continued over six days of microbial challenge (i.e., until the day before sacrifice).

### Experimental Mouse Model

Immunocompetent C3H male mice (22–29 g, Charles River Laboratories, Wilmington, MA) were studied. Plasma ammonia concentrations were assessed in uninfected, non-immunosuppressed mice (n = 5), as well as uninfected mice administered the immunosuppression regimen for 13 days (n = 5). Pharmacologically immunosuppressed mice were challenged with *U*. *urealyticum* in U9 broth over six days by four routes: intratracheal (IT) challenge every other day (n = 13), IP challenge every day (n = 15), intramuscular (IM) challenge every day (n = 15), or a combination of IT challenge every other day and IP challenge every day (n = 20). For IT challenge, mice were anesthetized with ketamine/xylazine (90/10 mg/kg), placed in a vertical position, and 50 μl of *U*. *urealyticum* suspension placed into the trachea using a 22G curved gavage needle. Mice remained vertical for two minutes and were monitored until awake. For IP and IM challenge, 250 μl of *U*. *urealyticum* suspension was injected into the peritoneal cavity or caudal thigh muscle, respectively. A vehicle negative control group (n = 14) was identically IT/IP challenged with spent U9 broth (filtered through a 0.1 μm filter). To assess the effect of pharmacologic immunosuppression alone, immunocompetent mice (n = 5) were also challenged IT/IP with *U*. *urealyticum*. *U*. *urealyticum* challenge was administered at least six hours after administration of immunosuppressive agents.

### Measurement of Plasma Ammonia Concentrations

Mice were euthanized with CO_2_ asphyxiation 24 hours after the last IP, IM, or IT/IP challenge or 48 hours after the last IT challenge. Blood was collected via cardiac puncture and placed into 1.5 ml EDTA collection tubes. EDTA blood was immediately centrifuged at 8000 rpm for 3 minutes and plasma frozen at -80°C. Plasma ammonia concentrations were determined within 24 hours using a Vitros 350 (Ortho Clinical Diagnostics, Inc., Raritan, NJ).

### *U*. *urealyticum* Culture and Real-time PCR Detection

Lung tissue, thigh muscle tissue and cardiac blood were cultured for *U*. *urealyticum* in U9 broth at 37°C for five days. Whole lung and thigh muscle were crushed in sterile stomacher bags with 3 ml MicroTest M5 transport media (Remel, Lenexa, KS); 200 μl of tissue homogenate was cultured. Positive cultures were confirmed by plating to A8 agar and by real-time PCR performed as described previously [9], using a LightCycler 1.5 real-time PCR instrument (Roche Diagnostic Gmbh, Mannheim, Germany) with probe dye Red-640. Additionally, *U*. *urealyticum* was directly assayed in lung tissue, thigh muscle tissue, and cardiac blood by real-time PCR after total DNA extraction using the DNeasy Blood and Tissue kit (Qiagen, Valencia, CA).

### Statistical Analysis

Plasma ammonia concentrations between groups were compared using Wilcoxon ranks sum tests. All tests were two sided; p-values less than 0.05 were considered statistically significant. No adjustment for multiple comparisons was made due to the small sample sizes. Analysis was performed using SAS version 9.4 (SAS Inc. Cary, NC).

## Results

### Plasma Ammonia Concentrations

The plasma ammonia concentrations of normal (non-immunosuppressed, uninfected) C3H mice (n = 5) were 160–280 μmol/L. The plasma ammonia concentrations of uninfected immunosuppressed mice (n = 5) were 171–340 μmol/L. The range of plasma ammonia concentrations of immunocompetent mice challenged IT/IP with *U*. *urealyticum* (n = 5) was 99–212 μmol/L (median, 178 μmol/L). The range of plasma ammonia concentrations of pharmacologically immunosuppressed mice challenged with spent U9 vehicle IT/IP (n = 14) was 155–330 μmol/L (median, 209 μmol/L). There was no difference in the plasma ammonia concentrations in this group compared with the other three control groups (median 212 μmol/L, range 99–340 μmol/L, p = 0.60).

**Fig 1:**
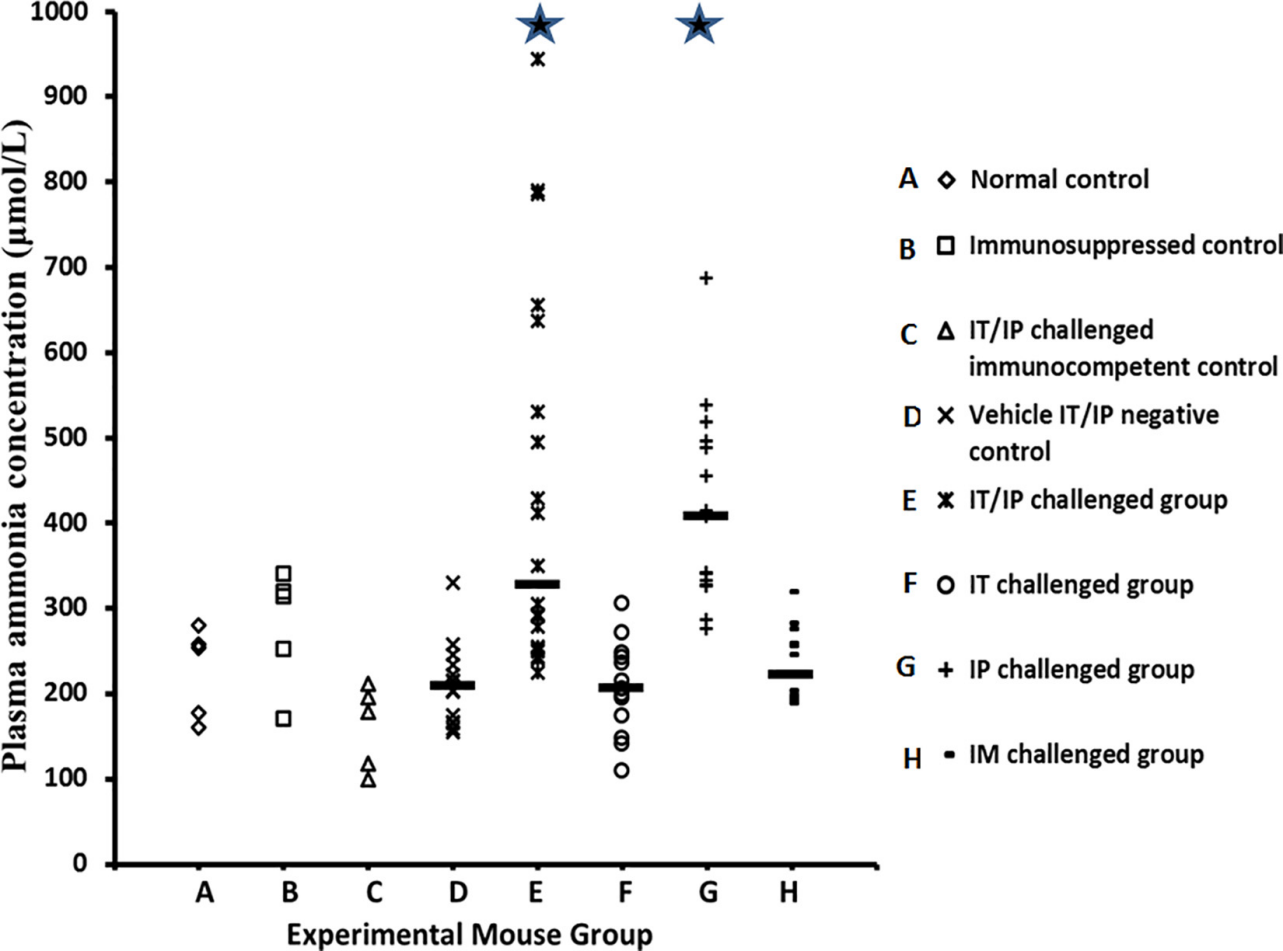
Plasma ammonia concentrations in the eight groups of experimental mice. The median of each immunosuppressed and infected group is shown by a short dash. Groups labeled by stars represent those with significantly elevated ammonia concentrations compared with group D, immunosuppressed mice inoculated with spent *U*. *urealyticum-*free U9 broth intratracheally (IT) every other day and intraperitoneally (IP) every day (P<0.001). Groups from left to right: **A)**. Immunocompetent and uninfected mice (n = 5). **B)**. Immunosuppressed but uninfected mice (n = 5). **C)**. Immunocompetent mice challenged with *U*. *urealyticum* IT every other day and IP every day (n = 5). **D)**. Immunosuppressed mice challenged with spent U9 broth without bacteria IT every other day and IP every day (n = 14). **E)**. Immunosuppressed mice challenged with *U*. *urealyticum* IT every other day and IP every day (n = 20). **F)**. Immunosuppressed mice challenged with *U*. *urealyticum* IT every other (n = 13). **G)**. Immunosuppressed mice challenged with *U*. *urealyticum* IP every day (n = 15). **H)**. Immunosuppressed mice challenged with *U*. *urealyticum* intramuscularly every day (n = 15).

Mice in the IT/IP challenge group (n = 20) had elevated plasma ammonia concentrations (median 328 μmol/L, range 225–945 μmol/L) compared to vehicle IT/IP negative control mice (p<0.001). Mice in the IT challenge group (n = 13) had similar plasma ammonia concentrations (median 206 μmol/L, range 110–306 μmol/L) to vehicle IT/IP negative control mice (p = 0.98). Mice in the IP challenge group (n = 15) had elevated plasma ammonia concentrations (median 408 μmol/L, range 276–687 μmol/L) compared to vehicle IT/IP negative control mice (p<0.001). Mice in IM challenge group (n = 15) had similar plasma ammonia concentrations (median 222 μmol/L, range 189–320 μmol/L) to vehicle IT/IP negative control mice (p = 0.15). A comparison of plasma ammonia concentrations for each group is shown in the Fig 1.

### *U*. *urealyticum* Culture and Real-time PCR

Culture and PCR results are shown in the Table 1. Uninfected immunosuppressed mice (n = 5), IT/IP challenged immunocompetent mice (n = 5), and spent U9 IT/IP vehicle control mice (n = 14) had negative cultures and PCR results. Among immunosuppressed mice challenged with *U*. *urealyticum* IT, IP, IM or IT/IP, all cardiac blood cultures and PCR results were negative, that is, no mice had detectable *U*. *urealyticum* bacteremia or DNAemia. In immunosuppressed mice challenged with IT *U*. *urealyticum*, 30% (10/33) of lung tissues were culture- or PCR-positive for *U*. *urealyticum*. All mice in the IP challenge group had culture- and PCR-negative lung tissues. All 15 immunosuppressed mice challenged with IM *U*. *urealyticum* (100%) had culture- or PCR-positive thigh muscle tissues.

**Table 1:** Experimental mouse group, interventions, culture and real-time PCR results.

Experimental Mouse Group	Number	Immunosuppressed	*Ureaplasma urealyticum* challenge	Blood	Lung	Thigh muscle
				Positive Mice (n) (Culture/PCR)		
Normal control (Immunocompetent, uninfected)	5	No	No	NA	NA	NA
Immunosuppressed control	5	Yes	No	0	0	NA
IT/IP challenged immunocompetent control	5	No	Yes	0	0	NA
Vehicle IT/IP negative control	14	Yes	Spent broth	0	0	NA
IT/IP challenge*	20	Yes	Yes	0	7 (0/7)	NA
IT challenge	13	Yes	Yes	0	3 (1/3)	NA
IP challenge*	15	Yes	Yes	0	0	NA
IM challenge	15	Yes	Yes	0	0	15 (13/11)

IT, intratracheal; IP, intraperitoneal; IM, intramuscular; NA, not applicable.

*: Groups with significantly elevated ammonia concentrations compared with vehicle IT/IP negative control (P<0.001).

Seven mice died prior to the blood and tissue collection. In the vehicle IT/IP negative control group, two died on day 10 because of anesthesia administered for IT challenge. In the IT/IP challenge group, one died on day 10, just after receiving immunosuppression; autopsy showed ascites. And, another died unexpectedly just before sacrifice on day 14. In the IT challenge group, one died on day 12 after anesthesia, while another died unexpectedly on day 14 just before sacrifice. In the IP challenge group, one died shortly after inoculation on day 8. Because of rapid coagulation of blood in deceased mice, their blood was not collected for ammonia determination. Beyond the seven mice described above, no other mice met criteria for humane endpoints requiring euthanasia prior to the experimental endpoint. The outcome of mortality was acknowledged as a possibility as part of our Institutional Animal Care and Use Committee approval, because hyperammonemia may lead to death, as can anesthesia and IT challenge.

## Discussion

Hyperammonemia syndrome is a previously-unexplained condition wherein lung transplant recipients develop progressive elevations in plasma ammonia concentrations within the first postoperative month, mostly within the first ten days [1–4, 6–8]. The majority develop cerebral edema, which causes mental status changes, and even seizures and/or coma. This condition can result in death. We recently reported an association between *U*. *urealyticum* or *U*. *parvum* and HS in lung transplant recipients [8]. Here, we have shown that *U*. *urealyticum* is sufficient to cause hyperammonemia in an immunocompromised experimental animal model.

*Ureaplasma* species, which belong to the class Mollicutes, are part of the normal genital tract flora. They lack a cell wall and do not grow on routine culture media, but can be isolated using specialized media containing urea. They produce large amounts of urease which hydrolyses urea to generate ATP [10]. In adults, *Ureaplasma* species have been associated with urogenital infections, while in neonates, they can cause invasive diseases, including pneumonia and bacteremia [11].

Herein, we have confirmed that *U*. *urealyticum* can cause hyperammonemia; immunosuppressed mice challenged with *U*. *urealyticum* IT/IP or IP had higher plasma ammonia levels (up to 945 μmol/L) than vehicle-challenged controls (p<0.001). This particular control was included to ensure that exogenous ammonia, present in the inoculated broth, would not yield hyperammonemia; we found that immunosuppressed mice challenged IT and IP with spent U9 broth did not have higher plasma ammonia concentrations than the other three control groups studied. As far as we know, immunocompetent adults infected with *Ureaplasma* species, with urethritis for example, do not develop hyperammonemia [11]. Consistent with this, immunocompetent mice not receiving pharmacologic immunosuppression, but challenged IT/IP with *U*. *urealyticum*, did not develop elevated plasma ammonia levels. This suggests that immunocompromised status per se may be important in the pathogenesis of *Ureaplasma-*related HS, and that *Ureaplasma-*associated HS may be an opportunistic infection-associated syndrome.

To our knowledge, this is the first model established to verify that *U*. *urealyticum* can cause hyperammonemia. In the model studied, the route of *U*. *urealyticum* challenge appeared to be important. This may be because the organisms live in or are absorbed through the peritoneum. Interestingly, in the IM-challenged group, all 15 thigh muscle tissues were culture- or PCR-positive for *U*. *urealyticum*, but no hyperammonemia was observed. Additional work is needed to clarify the exact role of route of challenge and specific immunosuppression in *U*. *urealyticum-*associated hyperammonemia. In lung transplant patients with HS, overt pneumonia (such as infiltrates on radiographic images or inflammatory lesions in tissue biopsy specimens) has not been reported, suggesting that although the lung may be infected, patients may not have classic manifestations of pneumonia. Due to the inability of the mice to tolerate IT-challenge more than every other day, we were unable to definitely establish whether IT challenge alone can cause hyperammonemia in our model.

There are several limitations to our study. We studied a type strain of *U*. *urealyticum*; we have anecdotally noted that different strains replicate at different rates and may produce different amounts of urease. Therefore, a clinical isolate may have resulted in differential virulence compared to that observed. In addition, there are two species of *Ureaplasma* associated with HS in lung transplant patients, and we only studied one of them. In future studies, it would be interesting to study *U*. *parvum* in the model described. IM challenge was included as this was model development and there is a prior *Ureaplasma* bacteremia model that was established in two-day-old mice via IM injection [12]; that there were no positive blood cultures or DNAemia was unexpected based on findings from the neonatal mouse study [12]. Whether the difference relates to mouse age, strain, or immune status, bacterial strain, culture or PCR methods, or other factors, remains to be determined. Considering that not every mouse in the IT/IP and IT challenge groups had positive lung cultures and/or PCR, some adult mice studied may have cleared *Ureaplasma* species. We only assessed the animals at a single time point, and may have missed documenting active *Ureaplasma* infection.

The pathogenesis of *Ureaplasma*-associated hyperammonemia remains to be determined. Specific factors (beyond exposure to the organisms) may predispose to *Ureaplasma* species-associated HS in lung transplant recipients. Previous studies showed that surfactant protein-A deficiency and host immune response to multiple banded antigens of *U*. *urealyticum* related to *Ureaplasma*-cidal activity [13, 14], and Beeton et al. showed that antibody mediated clearance is required for killing of certain serovars of *U*. *parvum* [15]. A recent article identified a single nucleotide polymorphism in toll-like receptor 6 associated with a decreased risk for *Ureaplasma* respiratory tract colonization and bronchopulmonary dysplasia in preterm infants [16].

The findings reported herein raise the intriguing hypothetical possibility that *Ureaplasma* species may be associated with unexplained hyperammonemia in patients other than lung transplant patients. A study evaluating host immune response in preterm neonates at risk of developing bronchopulmonary dysplasia showed that *Ureaplasma* DNA could be detected at low levels and decreased over time [17], raising the hypothetical possibility that *Ureaplasma* species could explain some cases of transient hyperammonemia of the preterm infant [18]. Unexplained hyperammonemia also occurs in intensive care unit patients, raising the hypothetical possibility that it too could relate to *Ureaplasma* species.

In conclusion, by establishing a novel experimental immunocompromised murine model and measuring plasma ammonia concentrations, we verified that *U*. *urealyticum* can cause hyperammonemia. Further research on pathogenesis, treatment, and host susceptibility may be studied in this new model. This work provides the impetus to determine whether *Ureaplasma* species might be associated with hyperammonemia in patient populations other than lung transplant recipients.
